# Directionally Oriented Reinforcements of Warp-Knitted Fabrics for Composite Preforms

**DOI:** 10.3390/ma17215221

**Published:** 2024-10-26

**Authors:** Katarzyna Pieklak

**Affiliations:** Faculty of Material Technologies and Textile Design, Textile Institute, Lodz University of Technology, S. Zeromskiego 116, 90-543 Lodz, Poland; katarzyna.pieklak@p.lodz.pl; Tel.: +48-42-631-33-38

**Keywords:** multiaxial knitted fabrics, knitted composite reinforcements, technical yarns, design of knitted fabrics, structural modification of knitted fabrics

## Abstract

This paper focuses on the development of a methodology for the directional structural modification of warp-knitted fabrics by sewing on carbon fiber tapes. Four-, five-, and six-axial geometric systems were designed to optimize the qualitative distribution of stresses on the surface of the tested product. Through a numerical experiment in the ANSYS environment, the impact of the change in the axiality of a textile structure on the mechanical properties of the modeled geometric configuration was assessed. This analysis was experimentally verified by measuring the multiaxial force distribution on the knitted surface, which demonstrated that Variant 7, with six axes 30° apart, was the most favorable.

## 1. Introduction

**Figure 1:**
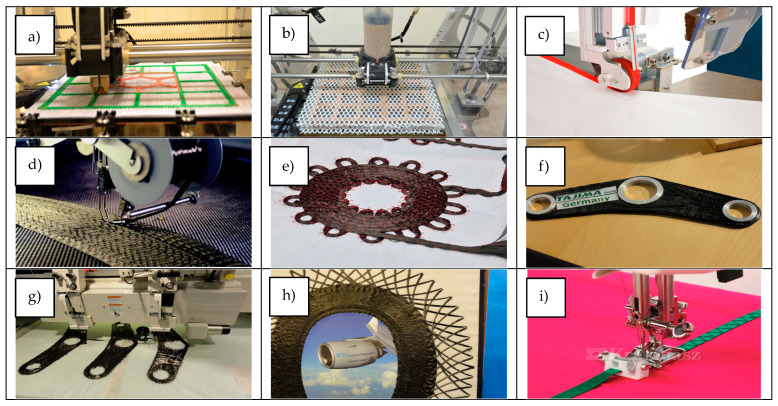
Available techniques for the directional reinforcement of fabrics: (**a**,**b**) additive technique [17,18]; (**c**) tape-sticking technique [19]; (**d**–**h**) tape sewing using the embroidery technique [20,21]; (**i**) tape sewing using sewing machines [23].

Directionally oriented structures (DOS) or non-crimp fabrics (NCF) are materials produced by introducing weft threads into the fabric structure via the warp knitting technique (stitch-bonding technique). The wefts in these fabrics can be arranged in up to four directions. The most popular reinforcement directions are 0°, 90°, and ±45° [1,2,3,4,5,6,7,8,9]. Such materials are commonly applied as composite reinforcements [8,10]. Technical yarns, made of glass, aramid, carbon, or basalt fibers, are frequently used for their production. A DOS warp-knitted fabric exhibits high tensile strength because of the directional layers built of straight yarns, unlike woven fabrics, which have less strength with the yarn arranged in a wave pattern. The layers of directionally oriented parallel yarns provide high dimensional stability. In addition, through appropriate stitch construction, DOS knitted fabrics are characterized by high resistance to shifting. The machines producing these technically knitted fabrics have high economic efficiency and a well-balanced cost–efficiency ratio [7,8,11,12,13]. To enable the functions performed by such textiles, we need to develop structures with specific mechanical parameters, which can be modified depending on the intended use of the product [3,14,15]. Such structural modifications to DOS fabrics can be achieved using various techniques and technologies (Figure 1).

One such method is the additive technique, in which reinforcements are applied using 3D printing on the surface of the textile structure, with a strictly oriented configuration [16,17]. Using this technique, we can determine the dispensing of the composite warp into the inner layer of the spatially knitted fabric [18]. Another way for reinforcing warp-knitted fabrics is fastening tape, such as that made of technical fibers, onto the fabric surface, in a strictly defined configuration [19]. Embroidery is yet another technology for the structural modification of products used in the textile industry, where an embroidery machine equipped with a special head sews strings or tapes onto the product [20,21]. This technique enables the precise application of reinforcing elements onto textile surfaces. Advanced programming environments allow for the creation of the track designs along which the reinforcement is sewn and the fabric’s density and number of layers, forming technical textiles with new desired mechanical properties.

Using the local reinforcement technique, we can place additional textile reinforcements on the textile’s surface in the form of a fiber path in the direction of maximum stress, significantly improving the specific strength. This approach often uses the embroidery technique to create technical embroidery that is characterized by additional, locally occurring, optimized reinforcement [22]. This technique can also be partially replaced by the use of a traditional sewing machines to create zig–zag stitches [23].

Scientific research shows the importance of the method of the distribution of the mechanical parameters in technical textile structures for many engineering applications. For instance, Gao Z. et al. clearly indicated the optimal tensile properties of multiaxial warp-knitted fabrics occur in the directions/axes of the textile reinforcement (yarn arrangement), e.g., in the case of three-axial structures, these are in the three axial directions, and these are in the four axial directions in four-axial structures [24]. In other research, Gao X. et al. stated that the yarn volume fraction and its orientation play important roles in the mechanical behavior of warp-knitted fabrics intended for technical applications. Solutions are available in which different row materials are combined in one structure, e.g., glass and carbon yarns or the type of stitch connecting subsequent layers of DOS material changes. The raw material has a significant impact on the mechanical parameters of non-crimp fabrics, whereas linking stitches made of warp-knitted yarn only stabilizes the technical yarns [25]. Moreover, Dexter et al. stated that the multiaxial knitting process enables the production of high-quality technical knitted fabrics [26].

Analyzing the literature on laminated materials made of continuous fibers, the authors in [27] stated that laminates are exceptionally strong and stiff in the direction of reinforcement. Meanwhile, in the other directions, laminates are much weaker because the load is carried by polymer matrices with less desirable mechanical parameters. In traditional textile laminate solutions, the tensile strength of high-strength fibers is 50 to 100 times higher than that of a typical polymeric matrix. When the composite is loaded, the fibers carry longitudinal tensile and compressive loads, while the matrix distributes the loads among the stressed fibers. Therefore, because the orientation of the fibers in the textile reinforcement has a direct impact on the mechanical properties, creating DOS materials with as many layers as possible oriented in different directions is ideal. Moreover, the authors also emphasized that the technology for obtaining multiaxial fabrics is relatively inexpensive [27].

Currently, engineering design strives to create structural elements, including textile-reinforced composites, that are lightweight while maintaining high strength parameters [28]. This type of approach is based on issues related to bionics, a broadly understood science inspired by nature or biologically inspired engineering. One principle of bionics is the imitation of forms and shapes inspired by solutions that exist in nature [29,30].

As Dickinson emphasized in [31], methods of producing various materials have become more sophisticated and advanced. Todoresin [32] cited that the arrangement of fibers in plants and trees is oriented according to a specific pattern, yielding desirable mechanical properties. One example is the coconut palm. The leaves that cover its trunk have a layered structure, in which the arrangement of fibers in the individual layers constitutes a biaxial structure. Moreover, coconut palm comprises three types of fibers with significantly different mechanical properties depending on their position on the palm trunk.

Another example of drawing inspiration from nature is the complex geometric structure of a spider’s web, which, in addition to its chemical composition, informs engineers on using spider-web-like designs for multiaxial structures, harnessing valuable features from the point of view of multidirectional stress distribution. While this approach yields newly developed solutions that may seem foreign in engineering science, they are very well-known in the organic world [32]. We are discovering the potential of imitating the functional principles of nature in the design of artificial structures to provide practical solutions to technical problems.

For example, Naderinejad et al. used the geometric structure of a spider’s web as inspiration for the design of vibration detection structures. They developed a large-area tactile sensor that could locate contact points by detecting vibrations. In addition, the authors analyzed how the structure of a spider’s web affects the efficiency of location detection, leading to the topological optimization of the web-like structure [33].

Sankaran et al. created bionically inspired multiaxial warp-knitted fabrics for the production of multifunctional textile reinforcements in lightweight composites. They designed and built a special manipulation unit for laying reinforcing threads integrated with a multiaxial warp-knitting machine. Their system also allowed the introduction of technical yarns into the textile structure; the position of these technical yarns in the knitting cycle can be modified depending on the specific application, obtaining textile preforms with improved mechanical properties [15].

Structures occurring in nature also seem to exhibit optimized weight. Moreover, the fibers constituting them are optimally oriented along the main direction of stress, and the number of these fibers is sufficient to ensure the necessary strength of a given “organic structure”. As previously mentioned, plants are made of fibrous materials characterized by various mechanical properties. For example, the stems of horsetails include differences in their individual layers, taking into account the optimal arrangement of fibers. This type of structure is a lightweight system with highly precisely defined bending stiffness and buckling stability using a minimal amount of material [34].

Nature provides technicians with a very wide field of extremely interesting models. Understanding the mechanisms occurring in the natural world can lead to significant technological progress by translating inspiration drawn from the natural environment into useful engineering technologies. By combining techniques of the design, modeling, analysis, and production of technical knitted fabrics, we can obtain textile composites with much better mechanical properties, customized for specific engineering applications.

The methods used to determine these parameters are, in most cases, based on the action of unidirectional force. However, such tests are not reliable because of their simplification; they do not take into account the anisotropic structure of textile reinforcements. In real conditions, these structures are subject to multidirectional forces. Therefore, the research methods used should reflect the actual operating conditions of such textile material as closely as possible. An example is to include such scientific considerations when determining the bending stiffness of knitted fabrics. The authors of [14] stated that a multidirectional measurement methodology, which allows the subsequent analysis of measurement results presented in the form of force distribution diagrams in many directions, may be useful in designing new products with specific mechanical parameters [14].

The literature contains concepts aimed at building devices for the multiaxial measurement of mechanical parameters of technical textiles. For instance, Vassiliadis et al. designed and built a new device for the multiaxial testing of textile materials. The part of the device on which the test sample was placed was circular with a diameter of 20 cm. This ring was divided into eight sectors, each of which served as a deformation sensor. This arrangement of the measuring setup allowed the authors to determine the mechanical parameters of the tested textiles along four axes, i.e., in eight directions every 45° [35].

In their study on the mechanical properties of PVC-coated biaxial warp-knitted fabric, Luo and Hu [36] also used a multiaxial tensile load measurement device. This instrument was developed at the Center for Textile Science and Technology of Minho University in Portugal. The measuring device has an octagonal structure; the diameter of the tested samples was 36 cm. Each sample was mounted on eight holders (arms), which received the applied tensile load, enabling measurements in eight directions every 45° [36].

Issues related to the design and construction of a measuring device for measuring multidirectional force distribution in textiles were also undertaken by scientists from the Lodz University of Technology in Poland. Their device was cylindrical with 24 handles placed around the circumference of the measuring part, 15° apart. These handles were connected to strain gauge sensors. Samples 20 cm in diameter were measured [37]. This device was used to conduct the experimental research presented in this publication (Section 4).

This work aims to develop a methodology for directionally reinforcing knitted fabrics by sewing on carbon fiber tape and to conduct numerical and experimental analysis of the surface stress distribution in knitted multiaxial textiles.

## 2. Research Material

### 2.1. Fabric Variants under Study

**Figure 2:**
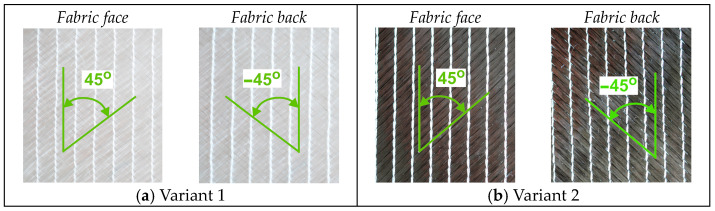
Biaxial DOS fabrics made of (**a**) glass fibers and (**b**) carbon fibers.

The research materials comprised seven variants of DOS technical fabrics, including two base structures (Variants 1 and 2) and five variants with structural modifications (Variants 3–7). First, an analysis was performed of two variants of biaxial fabrics with a surface mass density of 400 g/m^2^. These knitted fabrics were made of glass fibers with a linear density of 300 tex (Variant 1) and carbon fibers with a linear density of 506 tex (Variant 2) (Figure 2). Both fabrics were made of two sets of technical yarns arranged at 45° and −45° relative to the wales and interconnected via a pillar stitch.

### 2.2. Structural Modification of Biaxial DOS Fabrics

**Figure 3:**
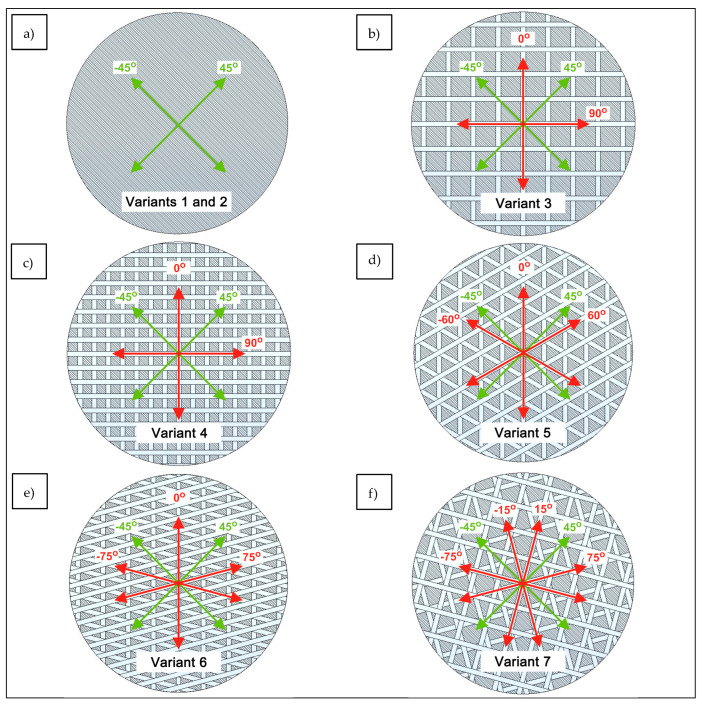
Structural modifications of DOS warp-knitted fabrics: (**a**) biaxial structure, (**b**,**c**) four-axial structures differing in reinforcement density, (**d**,**e**) five-axial structures differing in reinforcement inclination angle, and (**f**) six-axial structure.

To improve the mechanical parameters of the analyzed biaxial DOS structures, the directions were established for sewing on the carbon tape, as a result of which the analyzed glass fabric (Variant 1) underwent structural modification. The structure was built of two layers of glass yarns, arranged +45° and −45° relative to the wales and, thus, 90° from each other (Figure 3a). Such an arrangement of technical yarns in the product provides high strength in the yarn direction, i.e., ±45°. To visualize the changes in the product, the axes along which the threads of the base fabric are arranged are shown in green in the presented figures, while the directions of the added reinforcements are in red.

The mechanical parameters of the knitted fabric could be improved by expanding the structure using additional layers of yarns overlapping in the directions of the wales and courses, i.e., at 0° and 90°, giving the textile reinforcement the properties of a four-axial system (Figure 3b). By thickening the additional reinforcement along both the wales and the courses (Figure 3c), these structures can be further modified such that their strength properties are the same or even better compared to knitted fabrics made of a different fiber, e.g., carbon fiber (Variant 2).

A well-known and frequently applied geometric architecture is the “honeycomb structure”, i.e., a regular hexagon. This structure is a three-axial system built on the basis of equilateral triangles, in which individual axes are separated by 60°. In the case of the analyzed glass fabric, the application of additional tape in the aforementioned configuration provides the product with the properties of a five-axial reinforcement, in which the system transfers stresses at −60°, −45°, 0°, 45°, and 60° (Figure 3d). By changing the angles between the arms of the isosceles triangle, one can further modify the main directions of the loads carried within the structure (Figure 3e). In the case of technical textiles, including products intended for composite preforms, the main goal is the most uniform distribution of stresses in the entire system. Therefore, by increasing the number of axes in a DOS knitted structure, we can obtain unique geometrical configurations in which the materials in a two-dimensional system come close to gaining isotropic properties. Figure 3f presents a glass fabric reinforced with four additional axes, obtaining a six-axial structure in which the individual reinforcement axes are located at the following angles to the wales of the knitted fabric: −75°, −45°, −15°, 15°, 45°, and 75°.

## 3. Numerical Modeling of Mechanical Properties of Multiaxial DOS Knitted Fabrics

To verify the projected improvement in the stress distribution in the analyzed biaxial fabrics, this work carried out a numerical experiment using ANSYS software—2020 R1 release. For this purpose, a model of the geometrical structure of the designed textiles was created. Some simplifications were made to the cross-sections of glass and carbon yarns. They were assumed to have the form of cuboids of a certain thickness and width and made from raw materials with isotropic properties. The cross-sectional dimensions were 1.52 × 0.32, 1.92 × 0.31, and 6.00 × 0.16 mm for glass roving yarn, carbon roving yarn, and roving carbon tape, respectively. A value of 0.01 mm was taken for the distances between individual yarns and between subsequent planes of technical yarns.

With the use of the Tinius Olsen Testing Machine SAFQH5KL (Redhill, UK), Young’s moduli were determined for three types of yarns: glass and carbon yarns, used in Variants 1 and 2, and a 6 mm wide carbon roving yarn, used for modifying the glass fabric.

After the measurements, the determined Young’s moduli were as follows: for glass yarn, *E* = 7.481 GPa; for carbon yarn, *E* = 13.664 GPa; and for roving carbon tape, *E* = 40.729 GPa. Poisson’s ratios were taken from the literature: *ν* = 0.23 for glass fibers and *ν* = 0.32 for carbon fibers [38,39].

**Figure 4:**
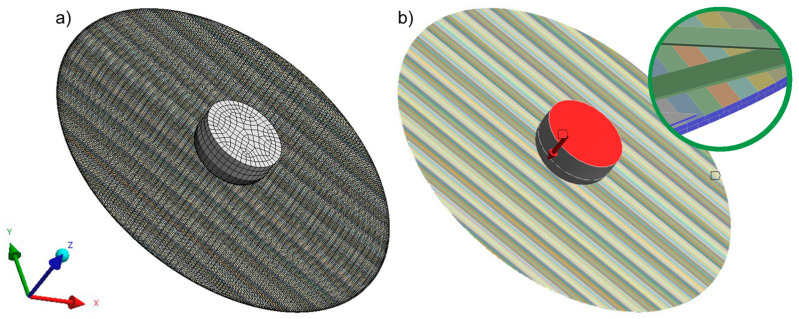
Preparation of numerical experiment: (**a**) finite element mesh and (**b**) loading and fixing of the model.

A finite element mesh was applied to the developed geometric models (Figure 4a). The sweep method was used as a division method, enabling a local increase in the mesh density in the contact area between the loading pin and the knitted fabric. A load of 196 N (20 kg) was applied to the upper surface of the loading mandrel (Figure 4b, red), and the entire textile structure was fixed by reducing all the degrees of freedom in the lateral planes around the designed composite reinforcements, i.e., at the edges (Figure 4b, blue). The bottom surface of the textile structure was movable—no degrees of freedom were removed.

**Figure 5:**
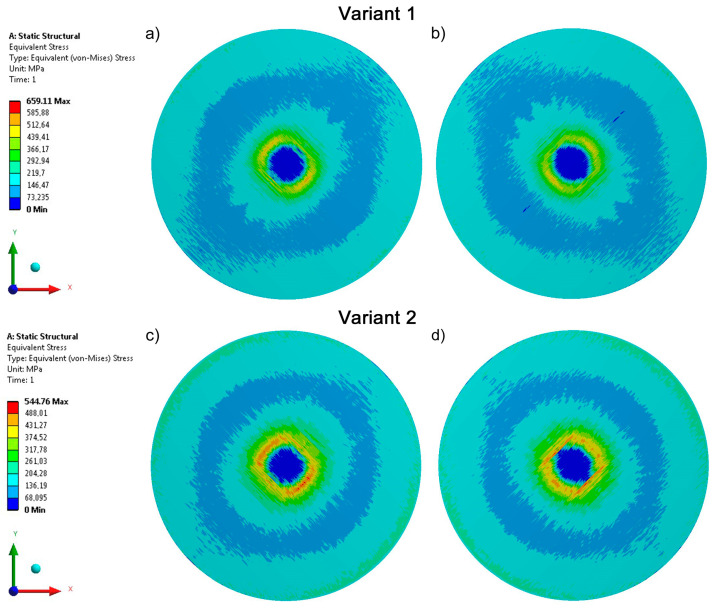
Stresses recorded for biaxial glass knitted fabric (Variant 1) and carbon knitted fabric (Variant 2) in the (**a**,**c**) top and (**b**,**d**) bottom layers.

**Figure 6:**
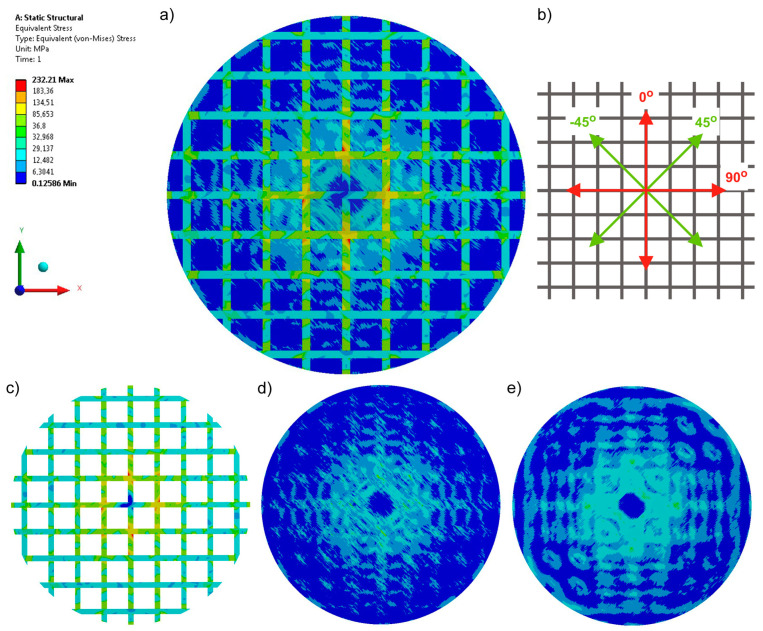
Equivalent stresses recorded for a four-axial knitted fabric (Variant 3): (**a**) stress in the whole structure, (**b**) configuration of axes in the product, (**c**) stress in the reinforcement made of carbon tape, (**d**) stress in the top layer, and (**e**) stress in the bottom layer.

**Figure 7:**
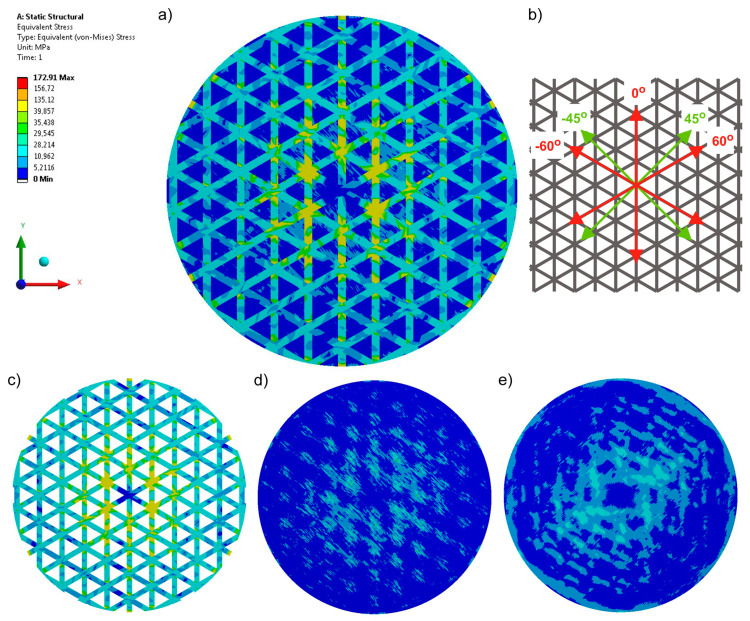
Equivalent stresses recorded for a five-axial knitted fabric (Variant 5): (**a**) stress in the whole structure, (**b**) configuration of axes in the product, (**c**) stress in the reinforcement made of carbon tape, (**d**) stress in the top layer, and (**e**) stress in the bottom layer.

**Figure 8:**
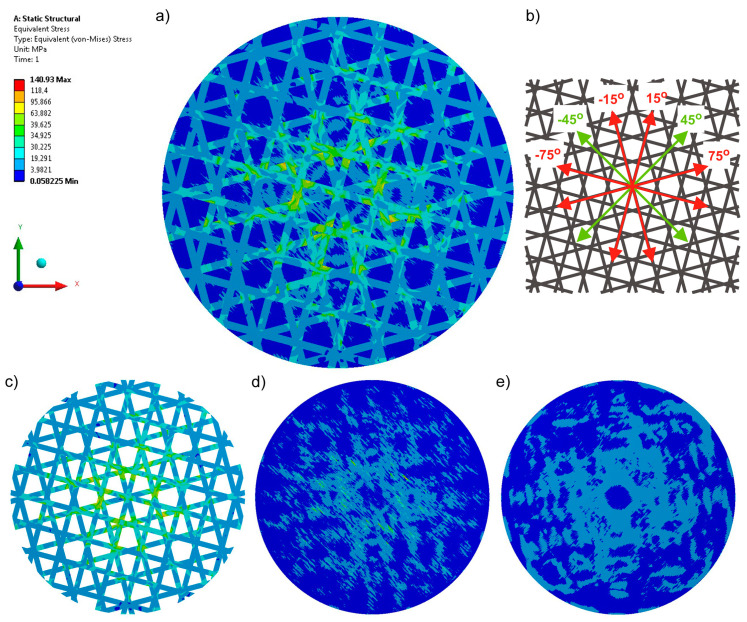
Equivalent stresses recorded for a six-axial knitted fabric (Variant 7): (**a**) stress in the whole structure, (**b**) configuration of axes in the product, (**c**) stress in the reinforcement made of carbon tape, (**d**) stress in the top layer, and (**e**) stress in the bottom layer.

The results obtained from the numerical modeling are presented in graphic form in Figure 5, Figure 6, Figure 7 and Figure 8.

An analysis of the distribution of equivalent stresses occurring in samples Variant 1 and Variant 2 (Figure 5) shows that they are strongly dependent on yarn direction (glass and carbon) in the knitted fabric structures. This characteristic is visible in both layers of the knitted fabrics, i.e., placed at ±45° relative to the wale direction. In addition, the maximum equivalent stresses in the carbon fabric are 17% lower than in the knitted fabric made of glass fibers.

Additional reinforcement of the biaxial DOS warp-knitted fabric (Variant 3) causes the equivalent stresses to appear in the directions of the wales and courses, i.e., at 0° and 90°. This phenomenon can be observed not only in the structure of the introduced reinforcement (Figure 6c) but also in the glass yarn layers (Figure 6d,e). In addition, the sewn-on carbon tape reduces the maximum stresses by approximately 75% compared to Variant 1 and by 57% compared to Variant 2.

In the case of the five-axial knitted fabric (Variant 5), the stresses are mainly transferred through the sewn-on carbon tape. The accumulation occurs in the place where force is applied. The presence of the three additional axes in the structure at 0° and ±60° (Figure 7c) also causes a change in stresses compared to the basic fabric made of glass yarns. In addition, maximum stresses were reduced by 73% compared to Variant 1, by almost 70% compared to sample Variant 2, and by 25% compared to Variant 3.

The last analyzed variant was a six-axial structure, in which four axes are introduced as reinforcement of the basic fabric at ±75° and ±15° (Figure 8, Variant 7). In this system, most of the stresses are also transferred through the sewn-on carbon tape, resulting in an even greater decrease in the occurring stresses (by almost 80% compared to Variant 1, by 74% compared to Variant 2, by 39% compared to Variant 3, and by 18% compared to Variant 5).

## 4. Experimental Verification of the Mechanical Properties of Multiaxial DOS Knitted Fabrics

**Figure 9:**
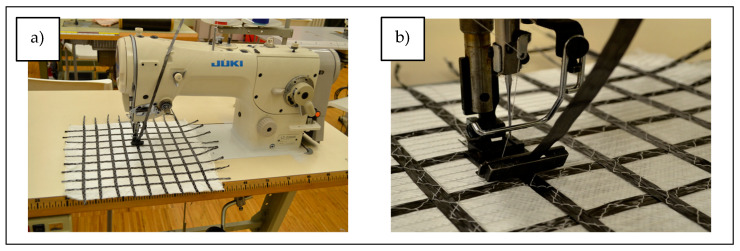
Juki lockstitch sewing machine, model LZ-2286N, sewing with zig–zag stitch: (**a**) setup for structural surface modification of a DOS biaxial glass knitted fabric; (**b**) equipped with a special presser foot guiding the tape.

According to the geometrical configurations of the multiaxial directions of stress distribution designed in Section 2.2, a tape made of carbon roving with a width of 6 mm and a linear density of 830 tex was fixed on the surface of a glass fabric (Variant 1). The carbon fiber tape was sewn on with a lockstitch sewing machine, Juki model LZ-2286N (Juki Corporation, Tokyo, Japan), using a three-step stitch (Figure 9). During the sewing process, a special presser foot was used to prevent the material from shirring.

**Figure 10:**
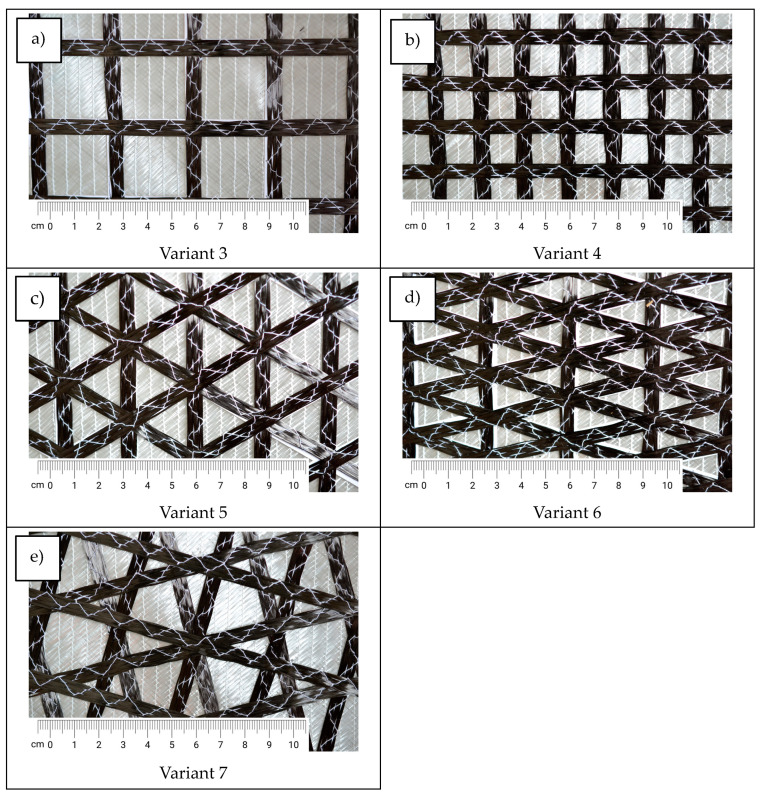
Obtained variants of multiaxial knitted fabrics: (**a**,**b**) four-axial knitted fabrics; (**c**,**d**) five-axial knitted fabrics; (**e**) six-axial knitted fabrics.

Figure 10 presents the obtained samples of multiaxial DOS fabrics, modified by sewing roving tape on one side.

For the obtained variants, the thickness and surface density were determined, and the results are summarized in Table 1.

To verify the distribution of forces in the developed structures experimentally, tests were carried out according to a measurement method of multiaxial stress distribution developed by Prof. Marek Snycerski [37]. The research was conducted at the Institute of Architecture of Textiles, Lodz University of Technology.

**Figure 11:**
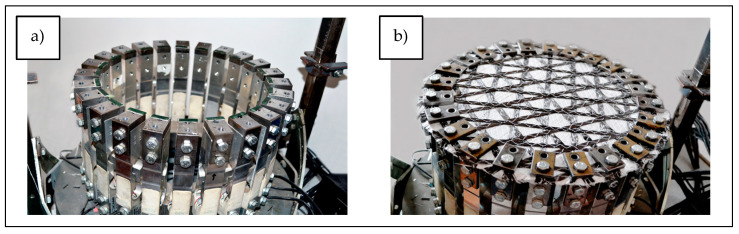
Instrument for measuring multiaxial stress distribution: (**a**) location of the handles; (**b**) fixing of the sample.

The measuring instrument is circular, with 24 handles equipped with strain gauges placed around it (Figure 11a), and 27 cm diameter samples were fixed on the upper part of the device (Figure 11b).

**Figure 12:**
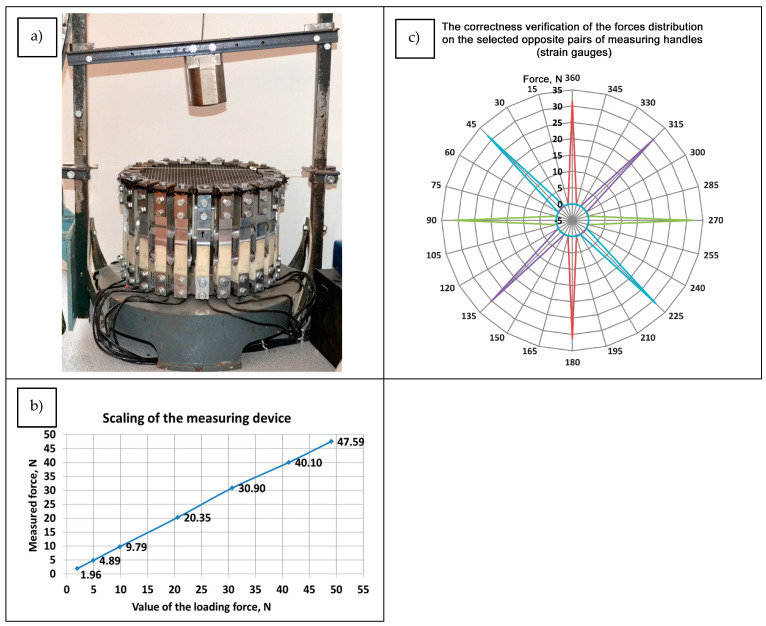
Experimental research on multiaxial force distribution on the surface of technical textiles: (**a**) measuring instrument; (**b**) calibration chart; (**c**) verification graph of the force distribution on opposite pairs of measuring handles.

The tested textile material was loaded on the central part of the sample with the following forces: 34, 49, 74, 98, 147, and 196 N (corresponding to 3.5, 5, 7.5, 10, 15, and 20 kg, respectively) (Figure 12a). Signals from the handles equipped with measuring sensors were registered using a DAS1202 measuring system. Before taking the measurements, a scaling procedure was carried out (Figure 12b), and the correctness of the force distribution on opposite pairs of measuring handles (strain gauges) was verified (Figure 12c).

**Figure 13:**
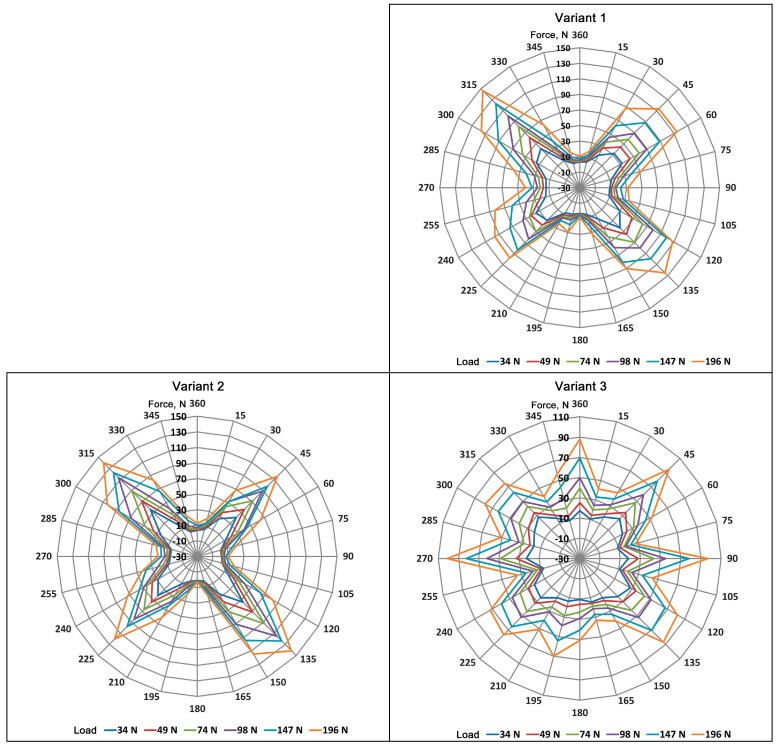

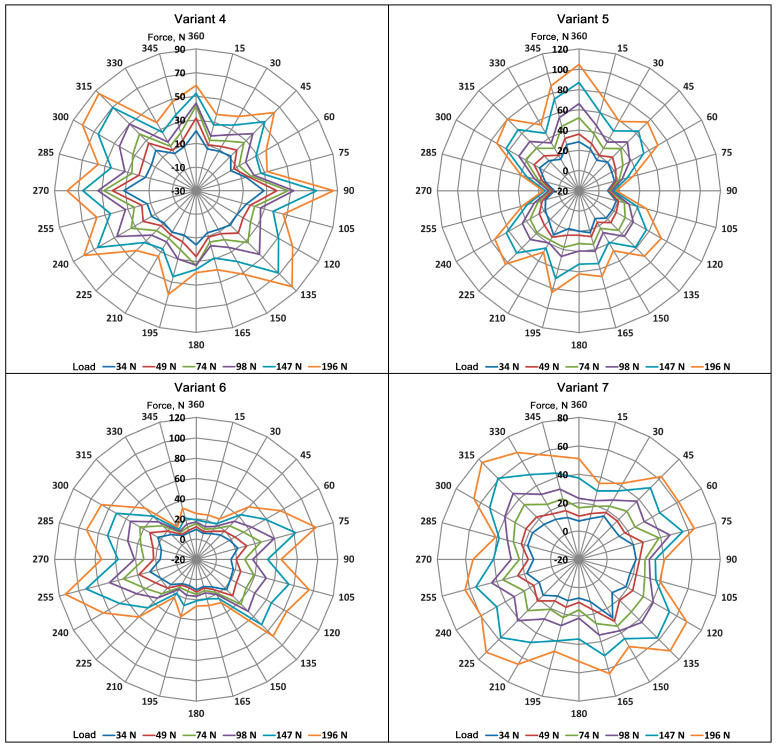
Charts of force distributions on the surface of the analyzed DOS multiaxial fabrics.

The obtained measurements for all seven variants of fabrics are graphically depicted with radar charts (Figure 13).

Based on the registered forces distribution in the surface of the analyzed multiaxial DOS knitted fabrics, in the case of Variants 1 and 2, in which the yarns forming the structures were arranged in two layers at +45° and −45° relative to the wales, the textiles exhibited strong orthotropic properties resulting from their construction. This was confirmed since maximum forces were registered by the sensors located in the device at the following angles: 45°, 135°, 225°, and 315°.

Modifying the knitted structure by increasing the number of axes from two to four, obtained by sewing on the carbon tape at 0° and 90° (Variants 3 and 4), resulted in forces in the directions of the added reinforcement. As a result, the qualitative distribution of forces in the fabric surface became more uniform compared to Variants 1 and 2. This is shown by the forces recorded at the measuring handles placed at 45°, 90°, 135°, 195°, and 225° or 240°, 270°, 300°, −315°, and 360°, which overlap with the axes of the product located at ±45°, 0°, and 90°. Of note, the density of the carbon tape reinforcement sewn onto the knitted fabric decreased the forces occurring in the sample.

Another modification, aimed at introducing three reinforcing axes, resulted in the accumulation of forces in the direction along the wales for Variant 5 and along the courses for Variant 6. The different force distributions observed in the analyzed samples resulted from the tapes sewn at angles different from that of the wales. For the Variant 5 sample, the forces were recorded on the sensors oriented at 45°, 60°, 120°, 135°, 195°, 225°, 240°, 300°, 315°, and 360°, which correspond to the configuration of axes in the product at 0°, ±45°, and ±60° angles. In Variant 6, significant forces were recorded on sensors oriented at 75°, 105°, 135°, 225°, 285°, and 300°, which correspond to the configuration of axes at ±45° and ±75°; in this sample, no significant effect was observed after introducing the reinforcement at 0°.

Variant 7 showed the most uniform force distribution on the surface of the sample because of the increased number of axes (up to six) and the angles between the individual axes, which were fixed at 30°.

In summary, the number of axes reinforcing the structure, their density, the angle between additional reinforcements and the wales in the fabric, and the angles between individual axes have significant impacts on force distribution in the tested samples.

## 5. Conclusions

1. Warp-knitted technical DOS fabrics are an increasingly popular group of textiles used for composite reinforcements. Depending on the intended application of the reinforcement, different mechanical characteristics of the textile system are required.
2. This work presents the concept of the structural modification of knitted biaxial structures by reinforcing them with carbon tape. In total, five configurations of the reinforcement were designed, including four-axial structures with variable reinforcement densities, five-axial structures with axes of varying angles with respect to the wales in the fabric, and a six-axial structure with a fixed angle between the axes.
3. The finite element simulation confirmed that introducing a reinforcement in the form of a tape made of carbon fibers changes the stress distribution of the modeled samples because part of the stress is taken on by the reinforcements.
  - Concerning the initial variants of DOS warp-knitted fabrics with a biaxial structure made of glass yarns (Variant 1) and carbon yarns (Variant 2), Variant 2 exhibited 17% lower maximum equivalent stress values than Variant 1.
  - Creating a four-axial structure by strengthening it with carbon tape in the 0° and 90° directions (Variant 3) reduced the maximum equivalent stress by approximately 75% in relation to Variant 1 and by 57% in relation to Variant 2.
  - For Variant 5, a five-axis system with additional carbon tape reinforcement in the 0° and ±60° directions, the maximum equivalent stress was reduced by 72% compared to Variants 1 and 2 and by 25% compared to Variant 3.
  - In the six-axis structure, the maximum equivalent stress was reduced by 77% for Variants 1 and 2, by 39% for Variant 3, and by 18% for Variant 5.
4. To verify the model’s results, experimental research was carried out on seven DOS knitted fabrics. With the use of a lockstitch sewing machine, five knitted fabric variants were produced by sewing on a 6 mm wide carbon tape using a zig–zag stitch, according to the designed geometrical systems. The obtained knitted fabric variants were subjected to tests based on the original method of determining multiaxial stress distribution. Analysis of the obtained force distributions in the tested samples yielded the following results:
  - For all samples, the distribution of the recorded maximum forces coincided with the direction of the reinforcement. The average recorded maximum forces caused by a load of 20 kg were as follows: in the two-axial structures, 121 N for Variant 1 and 129 N for Variant 2; in the four-axial structures, 87 N for Variant 3 and 75 N for Variant 4; in the five-axial structures, 78 N for Variant 5 and 79 N for Variant 6; and for the six-axial structure, the distribution of the recorded maximum forces on the sample’s surface was the most uniform, with an average value of 66 N. Therefore, the six-axial structure’s average maximum force was about 50% lower than in samples of Variants 1 and 2 and about 18% lower in relation to the remaining variants, i.e., Variants 3–6.
  - The analysis of the qualitative distribution of forces on the surface of the tested samples showed that the greatest uniformity was obtained for Variants 3, 4, and 7, where the latter was considered the most favorable in terms of its mechanical parameters, having the lowest recorded force values obtained as a result of the applied loading force. The samples in the two-axial system, i.e., Variants 1 and 2, were isotropic, while Variants 5 and 6 in the three-axial system were anisotropic.
5. In sum, the number of axes reinforcing the structure, their density, the angles between additional reinforcement and the wales, and the angles between individual axes have significant impacts on the mechanical properties of the samples.
6. The research presented in this work is a continuation of theoretical considerations on the oriented multiaxial reinforcing of warp-knitted fabrics using various methods, including additive techniques, sewing, and embroidery, as well as welding, sticking, and laminating technologies, which can be applied in the design and production of flat and spatial composite preforms.

## Figures and Tables

**Table 1:** Structural parameters of the obtained variants of knitted multiaxial composite reinforcements.

Variant	1	2	3	4	5	6	7
Surface density (g/m^2^)	400	400	490	552	553	606	556
Thickness (mm)	0.64	0.67	1.05	1.12	1.23	1.23	1.20

## Data Availability

The raw data supporting the conclusions of this article will be made available by the authors on request.
